# Supplementary material for: Tumor-Induced T Cell Polarization by Schwann Cells
Source: Cells. 2022 Nov 9;11(22):3541. doi: 10.3390/cells11223541 (PMC9688729; doi:10.3390/cells11223541)
Supplement: Supplementary file 1 [file cells-11-03541-s001.zip › cells-1984767-supplementary.pdf]

SUPPLEMENTAL MATERIALS

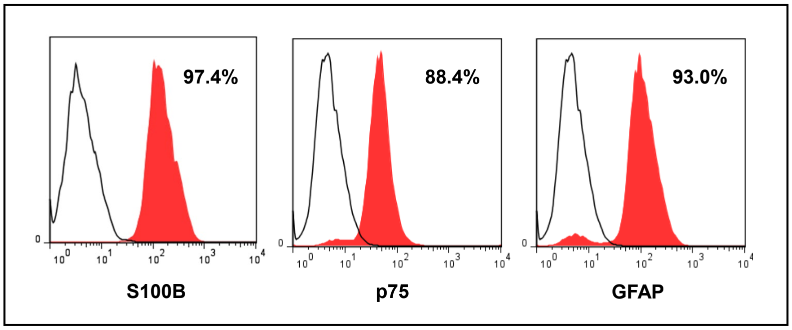

Figure S1. Verification of murine adult Schwann cell cultures.
Adult Schwann cell cultures were prepared from the sciatic nerves as described in M&M and their phenotype was characterized by flow cytometry using specific antibodies to neuroglia markers: S100B (S100 calcium-binding protein B), p75 (P75 nerve growth factor receptor, p75 NGF-R, also known as p75 neurotrophin receptor, P75NTR), and GFAP (Glial fibrillary acidic protein). All antibodies were from Abcam (Boston, MA, USA) and used as following: Alexa Fluor® 647 Rabbit monoclonal S100 beta (ab196175) (1:2000 dilution), APC rabbit anti-p75 NGF Receptor antibody (ab224996) (1:1000 dilution), and rabbit monoclonal to GFAP (ab207165 at 1/500 dilution; goat anti-rabbit IgG (Alexa Fluor® 488, ab150077) at 1/2000 dilution was used as the secondary antibody). Rabbit monoclonal IgG (ab172730) were used as isotype control. Results from a representative experiment are shown.

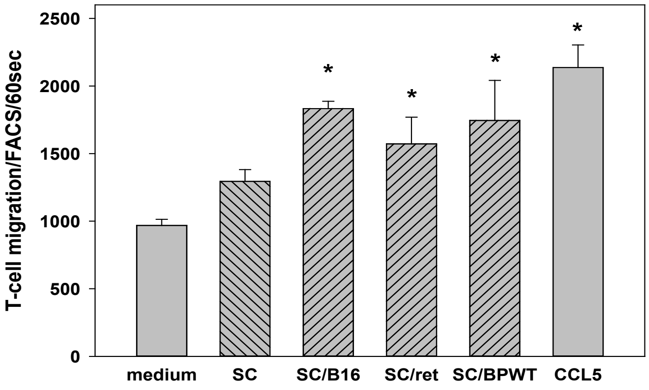

Figure S2. T cell chemoattraction by control and tumor-activated Schwann cells.
Adult SC were isolated from sciatic nerve of C57BL/6 mice, cultured and purified as described in M&M. SC were then co-cultured with medium (SC), B16 (SC/B16), ret (SC/ret) and BPWT (SC/BPWT) melanoma cell lines in inserts (2:1 cell ratio) for 48h and washed. Then, control and melanoma-pretreated SC (bottom chamber) were co-cultured with membrane-separated (5µm pore size) syngeneic splenic T cells (upper chamber) for 6h, and the number of transmigrated $CD3^+$ T cells (bottom chamber) was determined by flow cytometry for 60 sec (n=3 experiments; *, $p<0.05$ vs control SC; ANOVA). Results are shown as the mean ± SEM. Medium and CCL5 (20ng/ml) were used as negative and positive controls.

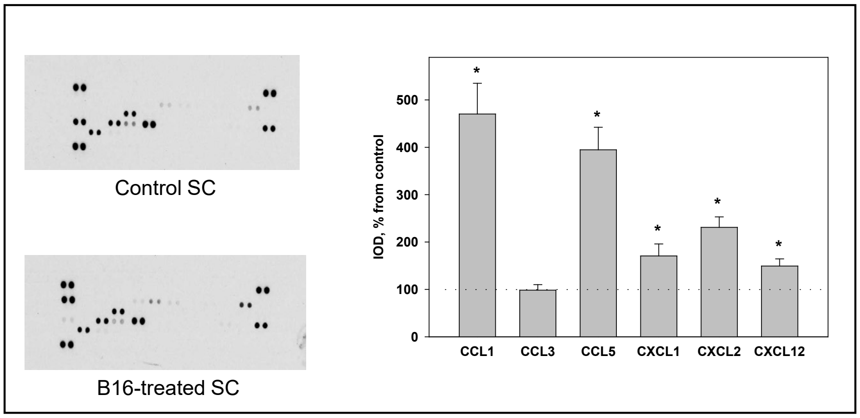

Figure S3. Proteome profiling of Schwann cells.
Lysates of control and tumor treated SC were used to quantify cytokines by Proteome Profiler Mouse Cytokine Array Kit (R&D systems, Minneapolis, MN, USA), a membrane-based sandwich immunoassay, according to the manufacturer's protocol. The nitrocellulose membrane containing different cytokine antibody spots was blocked and incubated with 50 µg of SC lysates overnight at 4°C. The membrane was washed and incubated with detection antibody cocktail for 1 h at room temperature. Then, the washed membrane was detected using peroxidase-conjugated streptavidin (left panels). The signal produced is proportional to the amount of analyte bound. Expression level was quantified using UN-SCAN-IT Gel Analyzer software (Silk Scientific, Inc., Provo, UT, USA). The cytokine spot intensities were measured with same area sizes and subtracted the background intensity of their adjacent area. The array data were quantitated to generate a protein profile and results are presented as average signal (pixel density) of the pair of duplicate spots representing each cytokine as Integral Optical Density (IOP) (right graph). The data presented are from three independent repeats and shown as the mean ± SEM. *, $p<0.05$, Student *t* test.

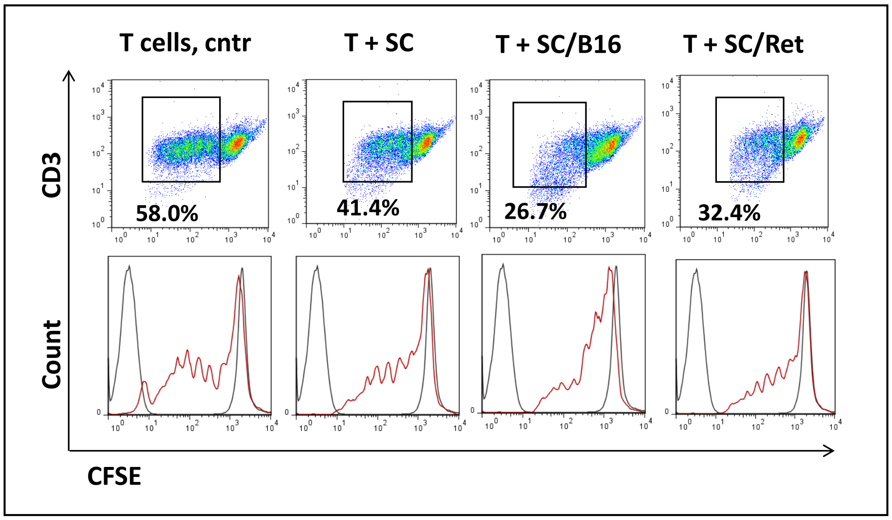

Figure S4. Suppression of T cell proliferation by tumor-activated Schwann cells.
Adult SC were isolated from sciatic nerve of C57BL/6 mice, cultured and purified as described in M&M. SC were then co-cultured with medium (SC) or with B16- or ret-conditioned medium (10% v/v) for 48 h and washed. Then, control and melanoma-pretreated SC were added to SFSE-labeled T cells that were activated by CD3-CD28-coated beads for 24 h. After 5-day co-culture, proliferation of CFSE-labeled CD3+ cells was assessed by tracing dye dilution by flow cytometry. Results from a representative experiment are shown.

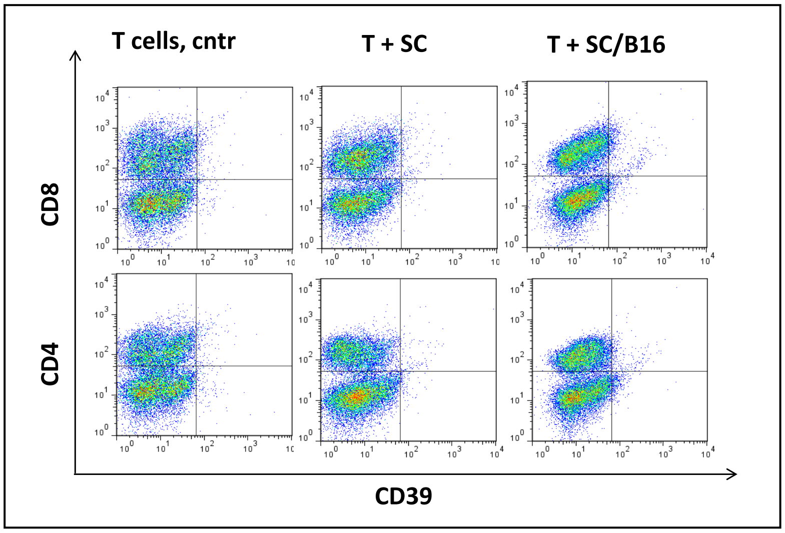

Figure S5. Schwann cells do not alter expression of CD39 in T cells.
Adult SC were isolated from sciatic nerve of C57BL/6 mice, cultured and purified as described in M&M. SC were then co-cultured with medium (SC) or with B16- and ret-conditioned medium (10% v/v) (SC/B16 and SC/ret) for 48 h and washed. Then, control and melanoma-pretreated SC were mixed with splenic T cells that were activated by CD3-CD28-coated beads for 24 h. After 5 days, T cells were stained with CD4, CD8 and CD39 fluorescently-conjugated antibodies and cell phenotype was evaluated by flow cytometry. T cells treated with medium served as a control (T cells, cntr). Results from a representative experiment are shown.

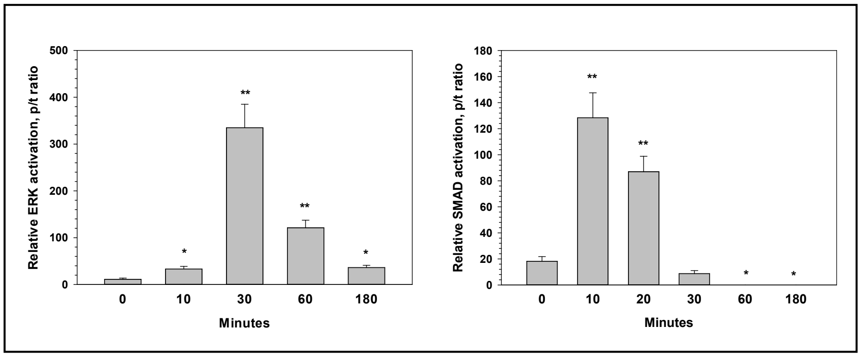

Figure S6. Tumor activated Schwann cells via SMAD2/3 and ERK1/2 pathways.
Adult SC were isolated from the sciatic nerve of C57BL/6 mice, cultured and purified as described in M&M. SC were then cultured with B16-conditioned medium (10% v/v) for 0-180 min, harvested, washed in cold medium, and their lysates were prepared for protein expression analyses. Expression of phosphorylated (p) and total (t) SMAD2/3 and ERK1/2 proteins in control and tumor-treated SC was determined by Western blot analysis. Cell extracts were subjected to SDS-PAGE electrophoresis and immunoblotting as indicated in M&M. The density of the bands was measured and quantified in pixel units using UN-Scan-It software. Relative expression of activated ERK1/2 (left panel) and SMAD2/3 (right panel) proteins was calculated as a ratio of phosphorylated to total (p/t) protein expression. Results are shown as the mean ± SEM of at least three independent experiments. *, $p<0.05$; **, $p<0.001$ (ANOVA).
